# Shen-Ling-Bai-Zhu-San for ulcerative colitis: Protocol for a systematic review and meta-analysis

**DOI:** 10.1097/MD.0000000000012337

**Published:** 2018-09-21

**Authors:** Long Yang, Yuanyuan Song, Pei Jin, Yueyang Liu, Yue Wang, Huixia Qiao, Yahui Huang

**Affiliations:** a Xi’an Hospital of Traditional Chinese Medicine, Xi’an; b Shaanxi land Enigineering Construction Group, Shanxi Province, China.

**Keywords:** protocol, Shen-Ling-Bai-Zhu-San, systematic review, ulcerative colitis

## Abstract

**Background:**

Ulcerative colitis (UC) is a chronic inflammatory bowel disease (IBD) at the colonic mucosa and submucosa. Shen-Ling-Bai-Zhu-San (SLBZS) is one of the most common formulations of traditional Chinese medicine (TCM) for the treatment of UC. However, its effects and safety remain uncertain. This protocol is described for a systematic review to investigate the beneficial effects and safety of SLBZS for UC.

**Methods:**

We will systematically search for eligible studies in PubMed, the Cochrane library, Embase, the Chinese Biomedical Literature Database (CBM), the China National Knowledge Infrastructure (CNKI), and Wanfang Data (WAN FANG) until August 2018. The primary outcomes are the induction of remission and the maintenance of remission. The summary results will be pooled using the random-effects model or fixed-effects model according to the heterogeneity of the included studies.

**Results:**

The results will be submitted to a peer-reviewed journal for publication.

**Conclusion:**

The conclusion of our systematic review will provide evidence to judge whether SLBZS is an effective intervention for patient with UC.

**PROSPERO registration number:**

PROSPERO CRD 42018100477.

## Abstract 2

Supplemental Digital Content is available in the text

## 1 Introduction

Inflammatory bowel disease (IBD) is a type of abnormal immune-mediated chronic and recurrent intestinal inflammation.^[1]^ With a variety of causes, IBD has a tendency of recurrence throughout life.^[2]^ Crohn disease (CD) and ulcerative colitis (UC) are the main disease types.^[3]^ UC is a chronic IBD at the colonic mucosa and submucosa, and its specific cause is still unknown.^[2]^ The main clinical manifestations of UC are recurrent diarrhea, mucus bloody stool, and abdominal pain.^[4]^ The onset of UC is often thought to be caused by a multifactorial interaction of environment, genetics, infection, and immunity.^[5,6]^ UC can occur at any age, but its onset is usually between 20 and 40 years old.^[7]^ The incidence of UC is reported to be 9 to 20 per 100,000 people every year, with a prevalence of about 150 to 300 per 100,000 people.^[7]^

The main purpose of UC treatment is to control the acute onset of the disease, heal the mucosa, maintain remission, reduce recurrence, and prevent complications.^[8]^ The drug treatment for UC is mainly anti-inflammatory drugs, including 5-aminosalycilate compounds, glucocorticoids, and immunosuppressive agents.^[9]^ Surgical removal of the colon should be performed if the effect of medical treatment is not satisfactory and the quality of life is seriously affected, or if the adverse reactions of hormones are too large to be tolerated.^[8]^

Traditional Chinese medicine (TCM) uses a variety of herbal mixtures to treat patients. These mixtures are referred to as formulas or “Fufang.”^[10]^ It is currently believed that the therapeutic effects exhibited by a lot of specific TCM therapies are regulated by multiple components.^[10]^ The presence of pharmaceutically active constituents has been demonstrated in these formulations, some of which have been reported to be effective in the treatment of a variety of diseases, including UC.^[11–13]^

Shen-Ling-Bai-Zhu-San (SLBZS) is one of the most common formulations of TCM for the treatment of UC.^[14]^ A number of studies published in Chinese medical literature have reported the efficacy and safety of SLBZS in treating UC.^[15–17]^ SLBZS is a famous classic formulation in *Taiping Huimin Heji Ju Fang* written by the Song Dynasty officials in 1078 AD.^[18]^ It is mainly composed of the following 10 kinds of TCMs, including *Panax Ginseng, Poria Cocos, Atractylodes Ovata, Dioscorea Batatasm, Coix Lachryma-jobi, Nelumbo Nucifera, Dolichos Lablab, Glycyrrhiza uralensis Fisch, Amomum Xanthioides,* and *Platycodon Grandiflorum*. On the basis of the theory of TCM, SLBZS has the function of supplementing spleen and is mainly used for weakness of the spleen and stomach.^[18]^ Studies have shown that SLBZS has a good clinical effect in the treatment of UC, and its mechanism may be related to inhibition of the formation of NLRP3 inflammasome and inhibition of inflammatory response.^[19]^

At present, there are many clinical trials reporting that SLBZS for the treatment of UC can improve clinical efficacy and reduce recurrence rate, but the sample size of each study is relatively small.^[15–17]^ As a result, the reports have large differences and weak stringency. Therefore, the international guidelines for the treatment of UC do not regard SLBZS as a reliable treatment. On the basis of this, the study systematically evaluates the clinical efficacy of SLBZS for UC using a meta-analysis method, so as to provide more evidence-based medical evidence for clinicians.

## 2 Methods

### 2.1 Inclusion criteria for study selection

#### 2.1.1 Types of studies

Randomized controlled trials (RCTs) will be included without restriction of publication type or language.

#### 2.1.2 Types of patients

Regardless of the subtype of UC, all participants diagnosed with UC will be concerned. There will be no restrictions on sex, age, ethnicity, economic status, or education.

#### 2.1.3 Types of interventions

Studies reporting any type of SLBZS treatment will be included. SLBZS could be used alone or combined with routine pharmacotherapy. Studies where the control group is different from the pharmacotherapy in the intervention group will be excluded. Control interventions will include no treatment, placebo control, routine pharmacotherapy, and other conventional treatments.

#### 2.1.4 Types of outcome measures

##### 2.1.4.1 Primary outcomes

The primary outcomes of this review will focus on the induction of remission and the maintenance of remission. The definitions employed in the primary studies will be accepted for these outcomes.

##### 2.1.4.2 Secondary outcomes

**Secondary outcomes are as follows:**
(1) improvement of clinical symptoms;
(2) changes in participant status as evaluated by quality of life;
(3) adverse events;
(4) cost (if available).

### 2.2 Search methods for the identification of studies

#### 2.2.1 Electronic searches

We will systematically search for eligible studies in PubMed, the Cochrane library, Embase, the Chinese Biomedical Literature Database (CBM), the China National Knowledge Infrastructure (CNKI), and Wanfang Data (WAN FANG) until August 2018. The reference list of relevant studies will be checked to identify additional studies. Search strategy of PubMed is shown in Appendix A.

#### 2.2.2 Searching other resources

Meanwhile, we also retrieve relevant documents by hand, such as replace and replenish some reference documents such as medical textbooks and clinical handbooks about the experiment; at the same time, we will contact with experts in the field and the writer to obtain important information that cannot be found from the retrieval.

### 2.3 Data collection and analysis

#### 2.3.1 Selection of studies

Two researchers will scan the titles and summary of the articles they get based on an inclusion criterion that is made previously to eliminate some uncorrelated documents; besides, for the documents that fit the inclusion criteria, the valuators will read the whole article to make sure if they meet a criterion and prepare to extract relevant information, check the result of the documents brought in. If it meets any diverges, the problem will be solved by consulting another researcher. The lacking information will be replenished by contacting with the writer of the original article.

#### 2.3.2 Data collection and management

Two researchers extract information from the documents that met the inclusion criteria, including disease diagnosis, comorbidity of disease, course of disease, severity of disease, sample size, age, gender, specific treatment plans, follow-up, outcome indicators, research results, and adverse events of intervention and control groups. When data are missing, wrong, or unclear, it shall be resolved through discussion within the group, contacting the author, or arbitration with a third party.

#### 2.3.3 Assessment of risk of bias in included studies

A tool introduced in the Cochrane Handbook for systematic reviews of interventions (V.5.1) will be used to assess a broad category of biases. This tool, include random sequence generation, allocation concealment, subjects and researchers blinded, outcome evaluation of blind method, the result data are incomplete and selective report results and other issues. Two reviewers will evaluate the methodological quality of the included trials independently. The results of the evaluation are low risk, unclear, and high risk. Inconsistencies can be resolved by discussion within the group, contacting the author to clear the details, or arbitration with a third party.

#### 2.3.4 Measures of treatment effect

The enumeration data are represented by relative risk (RR); measurement data will use mean difference (MD) and 95% confidence interval (95% CI) for each effect quantity.

#### 2.3.5 Dealing with missing data

As for the study lacking data, the researchers will attempt to obtain information by contacting the corresponding author. If fail, we will base our analysis on available data.

#### 2.3.6 Assessment of heterogeneity

The heterogeneity between the results included in the study was analyzed by the χ^2^ test.^[20]^ and the heterogeneity is quantitatively determined by combining with *I*^2^. If *I*^2^ is less than or equal to 50%, the statistical heterogeneity between the studies can be ignored. The fixed effect model is adopted to estimate the effect amount. If *I*^2^ is more than 50%, it is considered that there is great heterogeneity between the studies.

#### 2.3.7 Assessment of reporting bias

When there are more than 10 included studies, the publication biases are preliminarily determined by the symmetry of funnel plots. If the image is not clear, the Egger test is carried out with STATA 12.0 software for quantitative analysis.

#### 2.3.8 Data synthesis

A meta-analysis is carried out using RevMan 5.3 software (The Cochrane Collaboration, Oxford, England). If there is no statistical heterogeneity among the results of each study, fixed effect model will be adopted for meta-analysis. If there is statistical heterogeneity between the results of each study, the source of heterogeneity will be further analyzed. After excluding the influence of obvious clinical heterogeneity, the random effect model will be used for meta-analysis. If there is obvious clinical heterogeneity, subgroup analysis or sensitivity analysis are used to treat it, or only perform descriptive analysis.

#### 2.3.9 Subgroup analysis

Subgroup analysis will be performed on the basis of different interventions, controls, durations of treatment, and outcome measures. Adverse effects will be tabulated and assessed with descriptive techniques.

#### 2.3.10 Sensitivity analysis

To ensure robustness of the results, sensitivity analysis will be performed to eliminate the impact of low-quality studies, provided there is significant heterogeneity after subgroup analysis and input data validation. After the low-quality study is removed, the meta-analysis will be performed again. We will compare the results of these 2 meta-analyses and then decide whether to exclude low-quality studies based on sample size, evidence strength, and impact on aggregated effective size. However, if all of the included studies are at a high risk of bias, we will not conduct a sensitivity analysis.

#### 2.3.11 Ethics and dissemination

**Figure 1:**
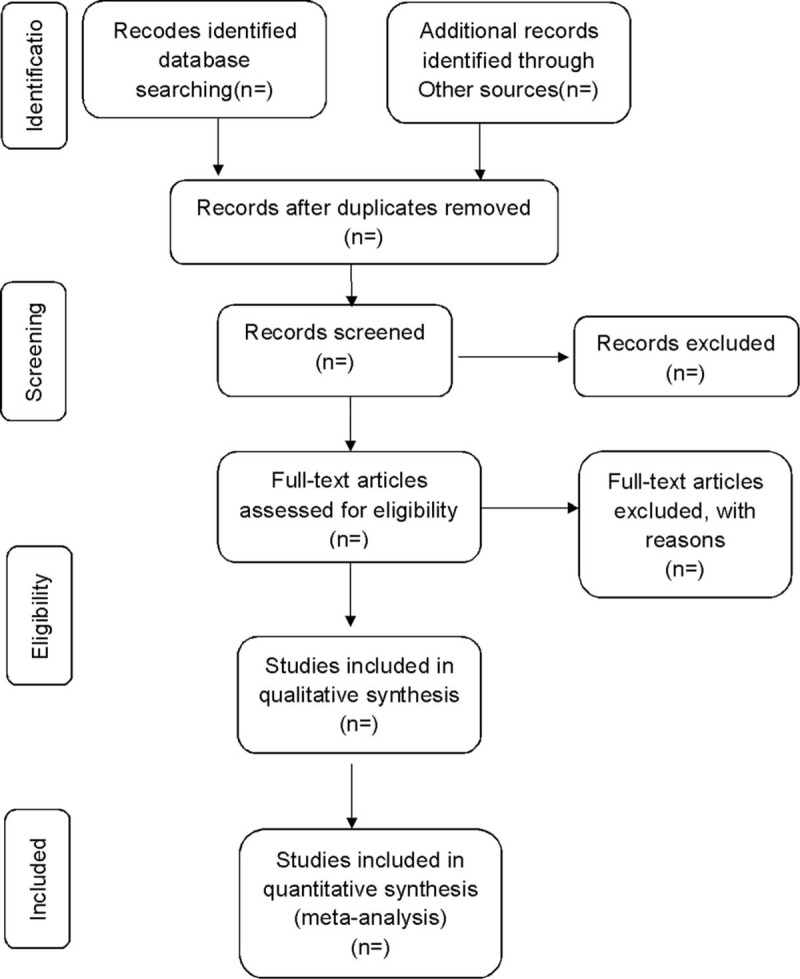
Flow diagram of study selection process.

This systematic review will not require ethical approval because there are no data used in our study that are linked to individual patient data. In addition, findings will be disseminated through conference presentations and peer-review publications (Fig. 1).

## 3 Discussion

**Figure 2:**
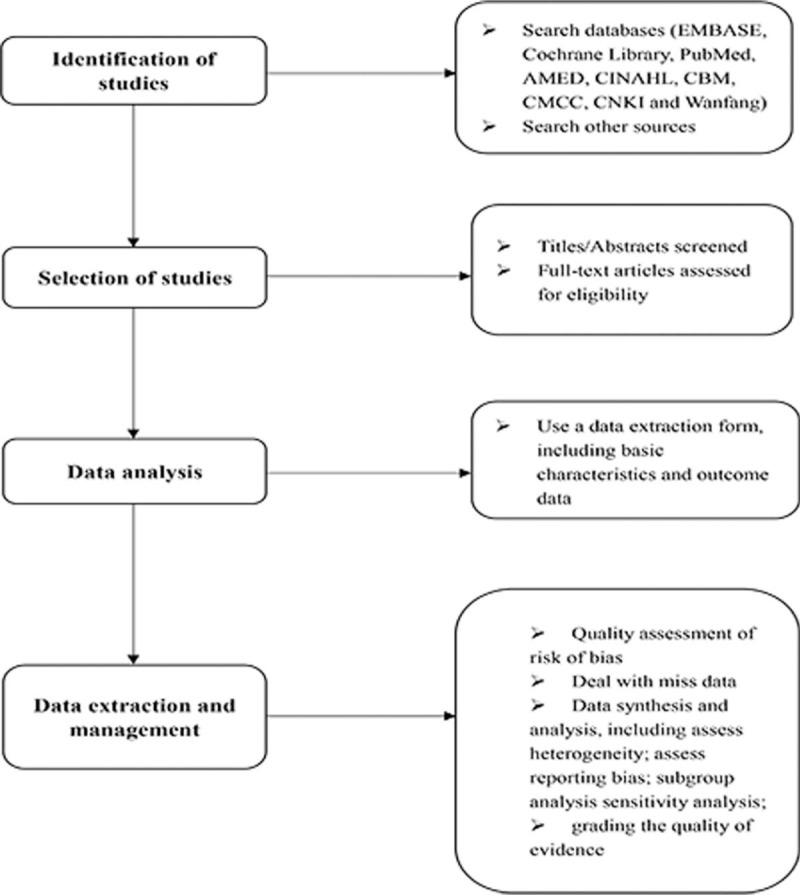
Flow diagram of the systematic review and meta-analysis.

Studies have shown that SLBZS can effectively alleviate the symptoms of UC (mainly diarrhea and abdominal pain).^[15–17]^ Nevertheless, there is no English version of the systematic evaluation of SLBZS for UC. The evaluation of the systematic review will be divided into 4 parts: identification, study inclusion, data extraction, and data synthesis (Fig. 2). We hope that this review will provide more convincing evidence to help clinicians make decisions when dealing with UC patients. There are also potential deficiencies in this study, and the different doses of SLBZS included in the trial and efficacy evaluation criteria for UC may result in significant clinical heterogeneity.

## Author contributions

**Data curation:** Long Yang, Yuanyuan Song.

**Formal analysis:** Long Yang, Yuanyuan Song.

**Methodology:** Yueyang Liu.

**Project administration:** Yueyang Liu, Yue Wang.

**Resources:** Yue Wang, Huixia Qiao.

**Software:** Huixia Qiao.

**Visualization:** Yahui Huang, Pei Jin.

**Writing – original draft:** Long Yang, Yahui Huang.

**Writing – review & editing:** Long Yang, Yahui Huang.

## Supplementary Material

Supplemental Digital Content
