# Supplementary material for: Shen-Ling-Bai-Zhu-San for ulcerative colitis: Protocol for a systematic review and meta-analysis
Source: Medicine (Baltimore). 2018 Sep 21;97(38):e12337. doi: 10.1097/MD.0000000000012337 (PMC6160248; doi:10.1097/MD.0000000000012337)
Supplement: Supplemental Digital Content [file medi-97-e12337-s001.doc]

**Appendix A.**

***Search strategy used in PubMed database***

#1 Shen-Ling-Bai-Zhu-San OR ShenLingBaiZhu

#2 Ulcerative Colitis OR inflammatory bowel disease

#3 Randomized controlled trial OR clinical study OR Clin-ical Trial OR Controlled study OR Controlled Trial OR Random*Control* study OR random* Control* Trial

#1 AND #2 AND #3
